# Training set optimization under population structure in genomic selection

**DOI:** 10.1007/s00122-014-2418-4

**Published:** 2014-11-01

**Authors:** Julio Isidro, Jean-Luc Jannink, Deniz Akdemir, Jesse Poland, Nicolas Heslot, Mark E. Sorrells

**Affiliations:** 1 Cornell University, Ithaca, NY USA; 2 Hard Winter Wheat Genetics Research Unit, USDA-ARS and Department of Agronomy, Kansas State University, 4011 Throckmorton, Manhattan, KS 66506 USA; 3 Limagrain Europe, CS3911, 63720 Chappes, France

## Abstract

****Key message**:**

**Population structure must be evaluated before optimization of the training set population. Maximizing the phenotypic variance captured by the training set is important for optimal performance.**

**Abstract:**

The optimization of the training set (TRS) in genomic selection has received much interest in both animal and plant breeding, because it is critical to the accuracy of the prediction models. In this study, five different TRS sampling algorithms, stratified sampling, mean of the coefficient of determination (CDmean), mean of predictor error variance (PEVmean), stratified CDmean (StratCDmean) and random sampling, were evaluated for prediction accuracy in the presence of different levels of population structure. In the presence of population structure, the most phenotypic variation captured by a sampling method in the TRS is desirable. The wheat dataset showed mild population structure, and CDmean and stratified CDmean methods showed the highest accuracies for all the traits except for test weight and heading date. The rice dataset had strong population structure and the approach based on stratified sampling showed the highest accuracies for all traits. In general, CDmean minimized the relationship between genotypes in the TRS, maximizing the relationship between TRS and the test set. This makes it suitable as an optimization criterion for long-term selection. Our results indicated that the best selection criterion used to optimize the TRS seems to depend on the interaction of trait architecture and population structure.

**Electronic supplementary material:**

The online version of this article (doi:10.1007/s00122-014-2418-4) contains supplementary material, which is available to authorized users.

## Introduction

Genomic selection (GS) emerged from the need to improve prediction of complex traits based on marker information (Meuwissen et al. 2001). The objective of GS is to improve the precision of selection by generating a genomic-estimated breeding value (GEBV) for selection candidates by simultaneously using genome-wide molecular marker information.

Genomic selection uses a training population set (TRS) of individuals that have been both genotyped and phenotyped to train a model that takes genotypic information from a candidate population of untested individuals and produces GEBVs for selection (Meuwissen et al. 2001). Genomic selection modeling takes advantage of the increasing abundance of molecular markers through modeling of many genetic loci with small effects (Whittaker et al. 2000; Xu 2003; Solberg et al. 2008; Habier et al. 2009; Zhang et al. 2011; Poland and Rife 2012). Over the last decade, simulation and empirical cross-validation studies in plants have shown GS to be more effective than strategies that use only a subset of markers with significant effects (Bernardo and Yu 2007; Heffner et al. 2009, 2011; Lorenzana and Bernardo 2009; Crossa et al. 2010; Jannink et al. 2010; Gonzalez-Camacho et al. 2012; Massman et al. 2013). Genomic Selection is superior to phenotype based-estimates for increasing gains per unit time even if both models show the same efficiency, because in principle, there is no need to record phenotypes of the candidates for the selection, hence shortening the length of the breeding cycle (Heffner et al. 2010).

The most commonly used methods to estimate GEBVs are (1) best linear unbiased prediction from mixed model analysis using a genomic-estimated relationship matrix (GBLUP) (Habier et al. 2007; Zhong et al. 2009) and (2) random regression-best linear unbiased predictions (RR-BLUP; Whittaker et al. 2000; Meuwissen et al. 2001). Genomic best linear unbiased prediction is a method that utilizes a genomic relationship matrix and potentially pedigree information to estimate the genetic merit of an individual. Elements of the genomic relationship matrix are estimated based on the proportion of the genome that two individuals share and predictions may be more accurate than those based on pedigree alone. For RR-BLUP, marker effects in the calibration set (CS) are estimated and then the GEBVs of the selection candidates are calculated by multiplying their marker scores by these estimates. Nevertheless, Habier et al. (2007) showed that both methods are equivalent.

The prediction accuracy of the GEBVs is normally evaluated using the correlation between the GEBVs and the true breeding values (TBV), *r* (GEBV, TBV). This correlation provides an estimate of selection accuracy and is directly related to selection response (Falconer and Mackay 1996), where *R* = *irσ*_A_, *i* = selection intensity, *r* = accuracy, and *σ*_A_ = the square root of the additive genetic variance (Falconer and Mackay 1996). Response to selection is important for determining gain per unit time and cost and for comparing breeding strategies. While new studies demonstrate that GS has great potential to increase rates of genetic gain, parameters determine its effectiveness for any specific breeding population. Factors that affect prediction accuracy include the number of markers used for estimating the GEBVs (Schaeffer 2006), trait heritability (Heffner et al. 2009), calibration population size (Jannink et al. 2010), statistical models (Heslot et al. 2012), number and type of molecular markers (Chen and Sullivan 2003; Poland and Rife 2012), linkage disequilibrium (Habier et al. 2007), effective population size (Daetwyler et al. 2008), relationship between calibration and test set (TS) (Albrecht et al. 2011; Clark et al. 2011, 2012; Pszczola et al. 2012) and population structure (De Roos et al. 2009; Saatchi et al. 2010, 2011; Windhausen et al. 2012; Guo et al. 2014).

In this study, we focus on the impact of population structure on GS accuracy. As a consequence of having different population genetic histories, distinct subpopulations could have differences in allele frequencies for many polymorphisms throughout the genome. If the populations have different overall values for the phenotype, any polymorphisms that differ in frequency between the two populations will be associated with the phenotype even though they are not casual or in strong linkage disequilibrium with casual polymorphisms (Pritchard and Donnelly 2001; Marchini et al. 2004; Price et al. 2010). Population structure is a key factor affecting predictions of breeding values with genomic models and could result in biased accuracies of genomic predictions (Saatchi et al. 2011; Riedelsheimer et al. 2013; Wray et al. 2013). Accordingly, population structure needs to be taken into account because it could lead to unrealistic assessments of accuracy (Riedelsheimer et al. 2013; Windhausen et al. 2012) and preferential selection of individuals within a single subpopulation, which would result in a loss of diversity in the breeding program.

Recently, the design of the TRS has attracted much interest in both animal and plant breeding, since it is critical to the accuracy of the prediction models. Knowing the predictability of a model is one of the key elements for a better allocation of resources in plant breeding, especially due to the high costs of phenotyping. Several studies have noted that the accuracy of genomic predictions is highly influenced by the population used to calibrate the model (Habier et al. 2007, 2010; Clark et al. 2011, 2012; Saatchi et al. 2011; Albrecht et al. 2011; Pszczola et al. 2012). Larger TRSs tend to increase accuracy but simulations suggest that, in some cases, small TRSs can be just as accurate (Habier et al. 2009). Generally, larger TRSs are required for traits controlled by more genes with smaller effects (Goddard and Hayes 2009). From the mixed model framework, given the trait heritability, marker data, and a TRS, it is possible to derive a measure of the quality of prediction for a set of genotypes. Two of those measures are the prediction error variance (PEV) and the coefficient of determination (CD). Rincent et al. (2012) used those criteria in an optimization procedure to choose a TRS of a given size in a maize diversity panel.

In quantitative genetics the PEV is central to the calculation of accuracies of estimated breeding values (Henderson 1975), to the restricted maximum likelihood (REML) algorithms for the estimation of variance components (Patterson and Thompson 1971), and to methods that restrict the variance of response to selection (Meuwissen and Woolliams 1994). The trends in genetic variance over time can be explored using breeding values and PEV of Mendelian sampling deviations (Lidauer et al. 2007). Choosing a TRS by seeking to minimize the PEV, however, may (1) result in the sampling of close relatives since the PEV does not take into account the genetic variance within the TRS (2) lead to TRSs that diverge between traits of differing heritability. To mitigate the first problem, Rincent et al. (2012) used the CD (Laloë 1993) that maximizes the expected reliabilities of contrasts between each selection candidate and the population mean. The CD can be defined as the squared correlation between the true and the predicted contrast of genetic values. It is a function of the PEV and of the genetic variance.

Rincent et al. (2012) proposed CDmean as a criterion to maximize the consistency of prediction for several CS sizes. This criterion gave higher predictions than random samples and the PEVmean, because CDmean took into account covariance among the TRS genotypes and avoided the selection of closely related individuals. When all the genotypes are independent, PEVmean and CDmean are equivalent (Laloë 1993).

The purpose of this study was to compare the performance of different optimization criteria, including one proposed by Rincent et al. (2012), in the presence of population structure and to evaluate how population structure interacts with these criteria in the choice of the TRS. During the different optimization methods, the genotypes for all the individuals in the CS are used, but the phenotypes were only required for individuals selected in the TRS at the model building stage. Finally, accuracies of the models were evaluated by calculating Pearson correlations between the predicted values and the observed phenotype values in the TS.

## Materials and methods

### Genetic dataset material

#### Wheat dataset

A population of 1,127 soft winter wheat varieties and F_5_—derived advanced breeding genotypes resulting from many different crosses in the Cornell University Wheat Breeding Program (Ithaca, NY) were analyzed in this study. Lines were genotyped with 38,893 genotyping-by-sequencing (GBS) markers (Table 1). Information about the construction and elaboration of the GBS libraries can be found in Poland and Rife (2012) and the latest updates on the GBS approach for wheat can be found on the website http://www.wheatgenetics.org/research). In summary, the GBS libraries were constructed in 95-plex using the P384A adaptor set. Genomic DNA was co-digested with the restriction enzymes PstI (CTGCAG) and MspI (CCGG) and barcoded adapters were ligated to genotype samples. Samples were pooled by plate into a single library and polymerase chain reaction amplified. Each library was sequenced on a single lane of Illumina HiSeq 2000 (Cornell Life Science Core Laboratory Center). Missing marker values were imputed using a multivariate normal (MVN)-expectation maximization (EM) algorithm (Poland and Rife 2012). The EM algorithm represents a general approach to calculating maximum likelihood estimates of unknown parameters when data are missing (Dempster et al. 1977). The EM imputation was designed for use with genotyping-by-sequencing (GBS) markers, which tend to be high density but have lots of missing data.

**Table 1:** Germplasm description summary and heritabilities values for each trait

Wheat		Rice
Population size	1,127	405
Markers	38,893 GBS	36,901 SNPs
Subpopulation	4	3
Environments	3	2
Years	6	2

Trait	*h* ^2^	Trait	*h* ^2^
YLD	0.79	FP	0.78
TWT	0.92	FT	0.85
LODG	0.78	PH	0.89
HD	0.94	PC	0.70
HT	0.95		

*GBS* genotyping by sequencing, *SNP* single nucleotide polymorphism, *h* ^*2*^ narrow sense heritability, *YLD* yield, *TWT* test weight, *LODG* lodging, *HD* heading date, *HT* plant height, *FP* florets per panicule, *FT* flowering time, *PH* plant height, *PC* protein content

Phenotypic data for five traits in the wheat dataset were analyzed: grain yield, test weight, lodging, heading date and plant height (Table 1). The experiments were carried out over 6 years from 2007 to 2012, with one location in 2007 and three locations per year from 2008 to 2012 near Ithaca, NY. Each location was arranged in an unreplicated augmented, row-column design (Federer 1956) with six check varieties replicated ten times each. First, in a mixed effect model an analysis was used to calculate best linear unbiased estimates (BLUEs) of locations and year effects (Mohring and Piepho 2009) and BLUPs for the genotypes (i.e., varieties or accessions) as random effects in ASRmel-R (Gilmour et al. 1995). Subsequently, these BLUPS were used for model building and the calculation of the accuracies of the models.

#### Rice dataset

The rice diversity panel consisted of 413 diverse accessions of inbred lines of rice (*O. sativa*) from 82 countries, including many landraces, representing all the major rice-growing regions of the world. This panel was genotyped with a 44-K chip (44,100 SNPs) and after filtering a total of 36,901 SNP markers were retained for genetic analysis (Ammiraju et al. 2006) (Table 1). Across the 12 chromosomes of rice, SNPs cover roughly 380 Mb of the genome at a density of about 1 SNP per 10 Kb. Each line was evaluated for important agronomic traits over 2 years with two replicates from 2006 to 2007. From this dataset, four different traits were selected (florets per panicle, flowering time in Arkansas, plant height and protein content) and phenotypic means of each inbred line across years and replicates were used for analysis (Table 1). All of the data from this study are publicly available at http://www.ricediversity.org and more details can be found in Zhao et al. (2011) and their supplementary data.

## Training set optimization methods

**Fig. 1:**
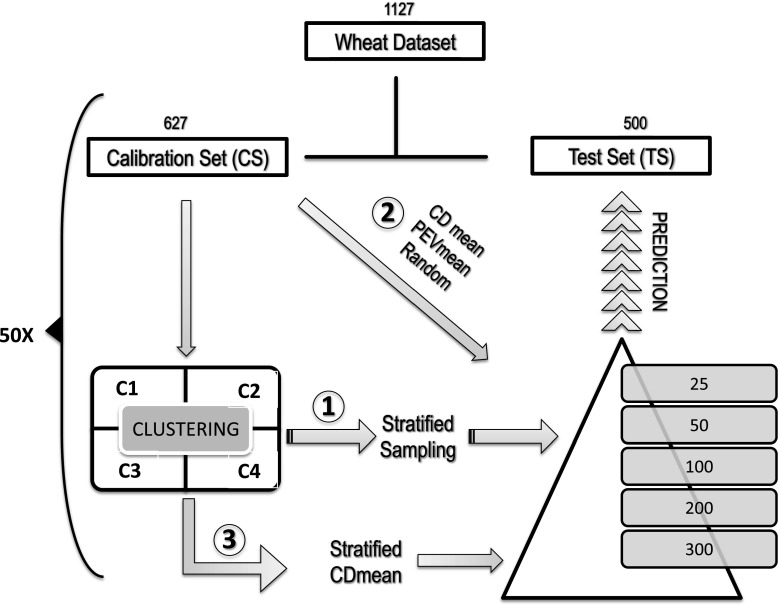
Example of optimization of training population set (TRS) scheme in the wheat dataset. The three methods are represented in *numerical circles*. *Number 1* represents the stratified sampling method, *number 2* the CDmean and PEVmean approaches and *number 3* the stratified CDmean. *C1*–*C4* acronyms indicate the number of cluster after analysis. More details about the specific methods can be found in the supplementary information in figures S3, S4 and S5. The optimization process was repeated over 50 runs and in a TRS size of 25, 50, 100, 200 and 300 genotypes

In this study, three different methods were developed to study the optimization of the TRS. Method 1 optimizes the TRS by stratified sampling, method 2 by CDmean, PEVmean and random sampling and method 3 combined previous methods to build the TRS. More details about the methods can be found in supplementary information S1, S2 and S3. Initially, the overall population was randomly divided into a calibration set (CS) and a test set (TS). Next, the CS was further divided into a training set population (TRS) and a remaining set (RS). Genotypes belonging to the TRS were used to create the prediction equation by a mixed model. The remaining genotypes in the RS were used to build the TRS in method 2 and method 3. The TS is the set of genotypes from the base population where predictions will be made, that is to say, where GEBVs are calculated to make selection. In our study, for all methods, the CS and the TS were randomly obtained from the overall population (Fig. 1, number 1). To ensure an accurate comparison among methods, the same CS and TS genotypes were used for each one of the TRS methodologies. In this study, we used datasets with information for all the phenotypes and genotypes. This allowed us to evaluate the accuracy of different TRS optimization methods. Nevertheless, in a real scenario the phenotypes are only available when the TRS is selected after the optimization process. Consequently, when selecting the TRS, only marker information was used. From the CS a subset of genotypes will be selected for phenotyping, which will build the TRS. The model built based on the phenotypes and genotypes in the TRS will be used to estimate the GEBVs for the genotypes in the TS. Here, we imposed the same population structure between CS and TS to avoid a potential prediction accuracy deflation that could arise when the TS population is not similarly stratified (Windhausen et al. 2012).

### Method 1—optimization based on stratified sampling by clusters

In this method, two random samples from the base population were taken to generate the Calibration set (CS) and the test set (TS). Then, a cluster analysis was run on the CS as follows: Genotypic markers were used to calculate the Euclidean distances between genotypes. Hierarchical clustering analysis using the Ward criterion (i.e., at each step the pair of clusters with minimum between-cluster distance are merged, generating clusters that were more equal in size) was applied to the Euclidean distance matrix. Principal components analysis (PCA) on genotypic data was used to visualize the structure of our populations. For the Cornell wheat program population, we selected four distinct subpopulations, based on genetic relationship and breeder´s knowledge. For the rice dataset we selected three distinct subpopulations. When the cluster analysis is obtained, the TRS is created by selecting a number of genotypes from each cluster proportional to the size of the cluster. Consequently, clusters with more genotypes will have a larger representation in the TRS than smaller clusters. With this method, we selected 25, 50, 100, 200 and 300 wheat genotypes and 25, 50, 100, 150 and 175 rice genotypes for the TRS. This methodology was repeated 50 times, and each time CS and TS were saved to assure a legitimate comparison among methods. The same CS and TS generated here were used to build the CS and TS for methods 2 and 3. Stratified sampling in clusters assured a high degree of genetic variability in the TRS, since each subpopulation was represented proportionally to its size. The optimization framework is shown in Fig. 1 number 1 and in supplementary information S1.

### Method 2—optimization criterion based on CDmean and PEVmean

The same CS and TS obtained in method 1 were used here to initiate the optimization. Firstly, a random sample of the target TRS size was obtained and the CDmean was calculated. Then, the optimization algorithm code provided by Rincent et al. was applied (Rincent et al. 2012) to our datasets. At each iteration, the algorithm randomly exchanged one genotype between the TRS and the set of RS genotypes. CDmean and PEVmean were then calculated. If the criterion was improved, the genotype exchange was accepted and otherwise rejected. The TRS optimization sizes sampled were the same as method 1. For each panel, 50 repetitions of the algorithm were performed and 2,000 iterations were needed to reach a plateau in the CDmean or PEVmean. The optimization framework is shown in Fig. 1 number 2 and in supplementary information S2.

#### PEV and CD optimization

A detailed description of the prediction model and optimization criteria was provided by Laloë (1993) and Rincent et al. (2012). We highlight here the model details and the calculation of PEVmean and CDmean. The criteria are based on the use of GBLUP (VanRaden 2008; Habier et al. 2007) to calculate the GEBVs.

GBLUP mixed model can be formulated as

$$\varvec{y} = X\varvec{\beta}+ Z\varvec{u} + \varepsilon$$

where ***y*** is a vector of phenotypes, ${\varvec{\upbeta}}$ is a vector of fixed effects (population mean in our case), **u** is a vector of random genetic values ${\varvec{\upvarepsilon}}$ is the vector of random residuals. *X* and *Z* are design matrices.

The variance of the random effects **u** is ${\text{var}}({\mathbf{u}}) = G\sigma_{\text{g}}^{2},$where ${\text{G}}$ is the genomic relationship matrix and *σ*_g_^2^ is the additive genetic variance in the panel. The variance of the residuals is ${\text{var}}(\varepsilon ) = {\mathbf{I}}\sigma_{\text{e}}^{2}$, where **I** is the identity matrix.

#### Criteria of optimization

The prediction error variance of ***u*** can be derived from the Henderson equation:

$$\left( {\begin{array}{*{20}c} {X^{{\prime }} X} & {X^{\prime } Z} \\ {Z^{{\prime }} X} & {Z^{{\prime }} Z + \lambda G^{ - 1} } \\ \end{array} } \right)\left( {\begin{array}{*{20}c} {\hat{\varvec{\beta }}} \\ {{\hat{\mathbf{u}}}} \\ \end{array} } \right) = \left( {\begin{array}{*{20}c} {X^{{\prime }} y} \\ {Z^{{\prime }} y} \\ \end{array} } \right)$$

where $$\lambda = {\raise0.7ex\hbox{${\sigma_{\text{e}}^{2} }$} \!\mathord{\left/ {\vphantom {{\sigma_{\text{e}}^{2} } {\sigma_{\text{g}}^{2} }}}\right.\kern-0pt} \!\lower0.7ex\hbox{${\sigma_{\text{g}}^{2} }$}}$$ is the ratio between the residual and the additive variances and $$G$$ is the genomic relationship matrix. Using the notation

$$\left[ {\begin{array}{*{20}c} {X^{\prime } X} & {X^{\prime } Z} \\ {Z^{\prime } X} & {Z^{\prime } Z + \lambda G^{ - 1} } \\ \end{array} } \right]^{ - 1} = \left[ {\begin{array}{*{20}c} {C_{11} } & {C_{12} } \\ {C_{21} } & {C_{22} } \\ \end{array} } \right]$$

$${\text{var}}\left( {{\mathbf{u}} |{\hat{\mathbf{u}}}} \right) = {\text{var}}\left( {{\hat{\mathbf{u}}} |{\mathbf{u}}} \right) = \left( {Z{\prime }MZ + \lambda G^{ - 1} } \right)^{ - 1} \;{ \times }\;\sigma_{\text{e}}^{2}$$

where *M* is a projector, orthogonal to the vector subspace spanned by *X* columns (*MX* = 0), $M = {\mathbf{I}} - X\left( {X^{\prime }X} \right)^{ - } X^{\prime }$ where $\left( {X^{{\prime }} X} \right)^{ - }$ is a generalized inverse of $X^{{\prime }} X$ (Laloë 1993).and therefore

$${\text{PEV}}\left( {{\hat{\mathbf{u}}}} \right) = {\text{var}}\left( {{\mathbf{u}} |{\hat{\mathbf{u}}}} \right) = {\text{diag}}\;C_{22} \;{ \times }\;\sigma_{\text{e}}^{2}$$

Contrasts allow us to compare the precision of comparisons between genotypes. The contrast will perform the comparison between genotype *i* and *j*, therefore for any contrast ***c*** of the predicted performances PEV can be calculated as:

$${\text{PEV}} = {\text{diag}}\left[ {\frac{{{\mathbf{c}}^{\prime } \left( {Z^{\prime } MZ + \lambda G^{ - 1} } \right)^{ - 1} {\mathbf{c}}}}{{{\mathbf{c}}^{\prime } {\mathbf{c}}}}} \right]\;{ \times }\;\sigma_{\text{e}}^{2}$$

where ***c*** is a vector of a particular linear combination whose elements sum to 0.

The aim in statistics is to minimize the error. Therefore, minimizing the mean of the PEVs of the contrast between each RS genotype and the mean of the CS panel is the goal of the optimization with PEV.

Laloë (1993) defined CD as the squared correlation between the true and the predicted contrast of genetic values.

The CD can be expressed as

$${\text{CD}} = R^{2} = \frac{{{\text{TSS}} - {\text{RSS}}}}{\text{TSS}} = \frac{{{\text{var}}\left( {\mathbf{u}} \right) - {\text{var}}\left( {{\mathbf{u}} |{\hat{\mathbf{u}}}} \right)}}{{{\text{var}}\left( {\mathbf{u}} \right)}} = \frac{{{\text{var}}\left( {{\mathbf{c}}^{\prime } {\mathbf{u}}} \right) - {\text{var}}\left( {{\mathbf{c}}^{\prime } ({\mathbf{u}} |{\hat{\mathbf{u}}}} \right))}}{{{\text{var}}\left( {{\mathbf{c}}^{\prime } {\mathbf{u}}} \right)}}$$

where TSS is the total sum of squares, RSS is the residual of sum of squares, **c** is the contrast between genotypes, ${\text{var}}\left( {\mathbf{u}} \right)$ is the total genetic variance and ${\text{var}}\left( {{\mathbf{u}} |{\hat{\mathbf{u}}}} \right)$ is the residual error variance or PEV. Making the corresponding substitution and calling

$$\left( {ZM^{\prime } Z + \lambda G} \right)^{ - 1} = \theta$$

$${\text{CD}}\left( {\mathbf{c}} \right) = \frac{{\sigma_{\text{g}}^{2} {\mathbf{c}}^{{\prime }} G{\mathbf{c}} - \sigma_{\text{e}}^{2} {\mathbf{c}}^{{\prime }} \theta {\mathbf{c}}}}{{\sigma_{\text{g}}^{2} {\mathbf{c}}^{{\prime }} {\mathbf{Gc}}}} = 1 - \frac{{\sigma_{\text{e}}^{2} {\mathbf{c}}^{{\prime }} \theta {\mathbf{c}}}}{{\sigma_{\text{g}}^{2} {\mathbf{c}}^{{\prime }} G{\mathbf{c}}}} = 1 - \frac{{\lambda {\mathbf{c}}^{{\prime }} \theta {\mathbf{c}}}}{{{\mathbf{c}}^{{\prime }} G{\mathbf{c}}}} = \frac{{{\mathbf{c}}^{{\prime }} \left( {G - \lambda \theta } \right){\mathbf{c}}}}{{{\mathbf{c}}^{{\prime }} G{\mathbf{c}}}}$$

and taking the diagonal elements of this matrix the CD can be expressed as

$${\text{CD}} = {\text{diag}}\left[ {\frac{{{\mathbf{c}}^{\prime }\left( {G - \lambda (Z^{\prime }MZ + \lambda G^{ - 1} )^{ - 1} } \right){\mathbf{c}}}}{{{\mathbf{c}}^{\prime } G{\mathbf{c}}}}} \right]$$

The CD corresponds to the expected reliability of the contrast between the predicted value of a given individual of the RS population and the population mean. The CD always lies within the unit interval. In this case, the optimization criteria will maximize the mean of the CD of the contrast between each non-phenotyped genotype (of the RS set) and the mean of the population (Rincent et al. 2012).

The relationship matrix used for the calculation of PEVmean and CDmean was the genomic relationship matrix (*G*). The relationship matrix is estimated as $G = \frac{{WW^{{\prime }} }}{f}$ where $W_{ik} = X_{ik} - 2p_{k}$ is the mean centered marker *k* for individual *i*, $p_{k}$ is the frequency of the 1 allele at marker *k* for the entire population, and *X*_*ik*_ denotes the number of minor alleles for the *i*th individual at marker *k*. Using a normalization constant of $f = 2\mathop \sum \limits_{k} p_{k} (1 - p_{k} )$, the mean of the diagonal elements is *1* + *f* (Endelman and Jannink 2012).

### Method 3—optimization criterion based on stratified sampling CDmean by cluster

The goal in this approach is to combine the strengths of methods 1 and 2. In this method, after the cluster analysis, the algorithm will create the TRS based on CDmean applied within each cluster. That is, rather than random stratified sampling, TRS members are selected within each cluster by the CDmean method. The same conditions on TRS size, number of iterations and repetitions were applied in this method as described in previous methods. The optimization framework is shown in Fig. 1 number 3 and supplementary information S3.

#### Heritability calculation and statistical software

Trait heritability was estimated across *e* environments and *r* replicates using a mixed model where environment was treated as a fixed effect and genotypes and genotype x environment interaction as random effects.

$$h^{2} = \frac{{\sigma_{\text{g}}^{2} }}{{\sigma_{\text{g}}^{2} + \frac{{\sigma_{\text{ge}}^{2} }}{e} + \frac{{\sigma_{\text{e}}^{2} }}{er}}}$$

where  $\sigma_{\text{g}}^{2}$, $\sigma_{\text{ge}}^{2}$, $\sigma_{\text{e}}^{2}$  are the additive, genotype by environment and residual variance components, *e* is number of environments and *r* is the number of replicates per environment.

All analyses were performed using R version 3.0 (2013). The package rrBLUP version 4.2 (Endelman 2011, http://cran.r-project.org/web/packages/rrBLUP/) was used to calculate GEBVs. We assessed the predictive ability of the models by the Pearson correlation coefficients between the GEBVs and the observed phenotypes in the TS (referred to here as accuracy). The training population set was also obtained by random sampling from the CS.

## Results

### Population structure

**Fig. 2:**
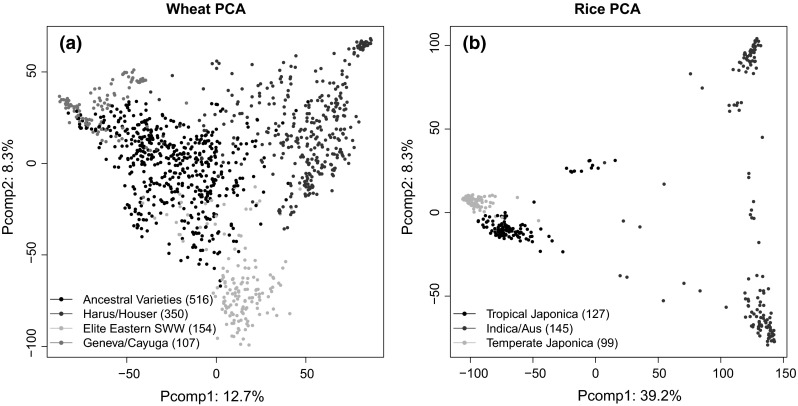
Plots of the first two principal components and the cluster analysis using *R* with 38,893 GBS and 36,901 SNPs markers on **a** wheat and **b** rice germplasm. Each *solid circle* represents a genotype and the * colors *indicate clusters membership. Legends summarize the distribution of the subpopulations for both germplasm. Number of genotypes per cluster is indicated in *parenthesis* (color figure online)

We performed PCA to summarize the genetic variation in both datasets. The analyses revealed structure in both populations (Fig. 2).

#### Wheat

Cluster analysis revealed that all of the clusters can be separated in the first two PC axes that accounted for 12.7 and 8.3 % of the genetic variance, respectively (Fig. 2a). The number of lines per cluster ranged from 107 to 516 (Table 2). The largest subpopulation size (516) corresponds to ancestral varieties from New York, Ontario, Ohio and Michigan, followed by genotypes derived from Harus/Houser/SuMei crosses. The third was formed by Elite Eastern soft winter from the eastern United States. Finally, genotypes from Geneva/Cayuga crosses (New York) formed the forth cluster. The structure explained in yield, test weight and height was 6.1, 5.7 and 8.2 % respectively. Lodging and test weight showed the highest proportion of variance explained by the clusters with 15.4 and 13.0 %, respectively (Fig. S4).

**Table 2:** Descriptions of wheat and rice clusters identified using hierarchical clustering model analysis

	Cluster	Number of lines	Origins^a^	Representative line
Wheat	C1	516	NY, Ont, OH, MI	Ancestral varieties
	C2	350	NY, Ont, China	Harus/Houser/SuMei
	C3	154	NY, MI, OH, IN, VA	Elite Eastern
	C4	107	NY	Geneva/Caledonia
Rice	C1	145	IN, CH, PH, BR,	Indica/Aus
	C2	127	US, BR, AR, CO, NI	Tropical Japonica
	C3	99	UE, JA, CH	Temperate Japonica

*NY* New York, *Ont* Ontario, *OH* Ohio, *MI*, Michigan, *IN* Indiana, *VA* Virginia, *IN* Indica, *CH* China, *PH* Philippines, *BR* Brasil, *US* United Stated, *AR* Argentina, *CO* Congo, *NI* Nigeria, *EU* European Union, *JA* Japan

^a^Based on Zhao et al. (2011)

#### Rice

The rice dataset is a very diverse panel from 82 countries and the analysis of population structure revealed three clear subpopulations. Clusters were separated in the first two PCs axes and accounted for 39.2 and 8.3 % of the total variance (Fig. 2b). Population sizes within clusters varied from 99 to 145 genotypes. A more detailed description of the accessions and geographical distribution of the rice germplasm can be found in Zhao et al. (2011). The proportion of the variance explained by the structure in the rice dataset can be found in supplementary information S5.

### Training set prediction accuracies

**Fig. 3:**
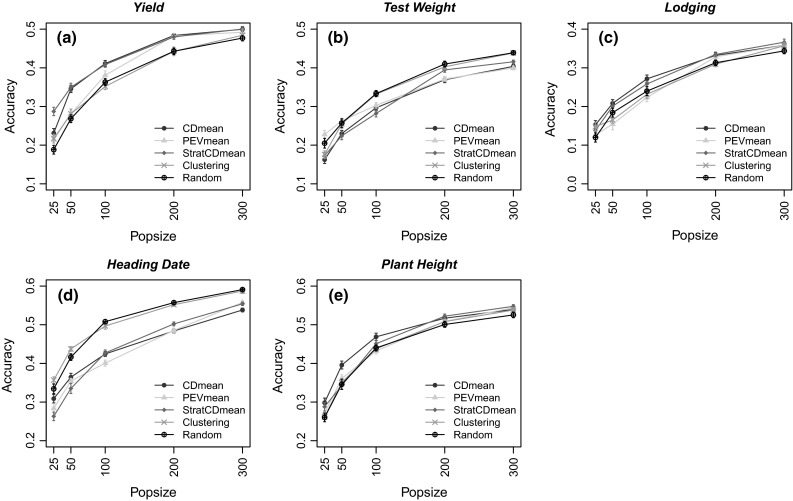
Accuracies of the predictions of the TS genotypes in the wheat germplasm. The calibration sets were defined by maximizing CDmean; minimizing PEVmean; maximizing CDmean within cluster; stratified proportional sampling and random sampling. Four different population sizes (25, 50, 100, 200 and 300) were used for the optimization algorithm in five different traits (**a** yield, **b** test weight, **c** lodging, **d** heading date, **e** plant height). *Standard error* is indicated for each point over the 50 runs. Optimization of CDmean, PEVmean and StratCDmean was made with the heritability measured for each trait in each germplasm (color figure online)

**Fig. 4:**
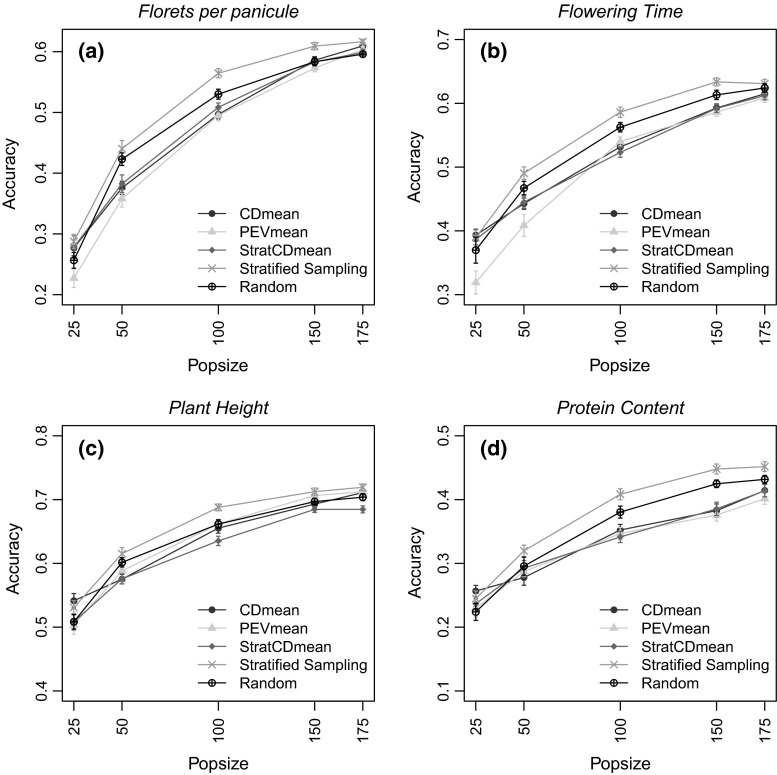
Accuracies of the predictions of the TS genotypes in the rice germplasm. The calibration sets were defined by maximizing CDmean; minimizing PEVmean; maximizing CDmean within cluster; stratified proportional sampling and random sampling. Four different population sizes (25, 50, 100, 150 and 175) were used for the optimization algorithm in four different traits (**a** florets per panicle, **b** flowering time, **c** plant height, **d** protein content). *Standard error* is indicated for each point over the 50 runs. Optimization of CDmean, PEVmean and StratCDmean was made with the heritability measured for each trait in each germplasm (color figure online)

Figures 3 and 4 show the accuracies of the predictions for the wheat and rice datasets. In general, accuracy values were lower in wheat. Accuracies ranged from 0.12 to 0.59 and from 0.20 to 0.72 in wheat and rice, respectively. In both populations, accuracies increased as the TRS size increased. Different heritability values and $\lambda$ did not change the patterns of accuracy for either dataset. Nevertheless, there were noteworthy differences in GS accuracies among TRS selection methods of optimization studied here.

In the wheat dataset, predictions using the CDmean and StratCDmean methods showed the highest accuracies for all the traits except for test weight and heading date. In general, CDmean and StratCDmean were not significantly different within traits, although some exceptions were found for the smaller TRS sizes (Fig. 3a, d). At the lowest TRS size, CDmean and StratCDmean showed the highest accuracies, with the exception of test weight and heading date. Usually, PEVmean showed lower accuracies than CDmean except for test weight where the PEVmean showed better accuracies in the two smallest TRS sizes. Stratified and random sampling showed similar patterns among traits. However, these methods showed the highest accuracies for test weight and heading date.

Predictions using the rice dataset showed higher accuracies than the wheat dataset overall even though the CS size (250) was smaller than for wheat (627) (Fig. 4). The stratified sampling method showed the highest accuracies for all traits. In this dataset, the calibration set of random sampling was always lower or equivalent to those obtained by stratified sampling for all traits. At the smallest population size, CDmean and StratCDmean showed the highest reliabilities but were not significantly different from stratified and random sampling. As the population size increased, their accuracies dropped below the stratified sampling approach, especially for plant height and protein content (Fig. 4c, d). Similar to the wheat population, PEVmean accuracies followed a pattern similar to CDmean and the differences between accuracies of CDmean and PEVmean were significant only for florets per panicle and flowering time at intermediate population size. For these traits, PEVmean showed the lowest accuracies (Fig. 4a, b). More information among accuracies across methods can be found in supplementary information S6 and S7.

### Selection optimization of the training sets

**Fig. 5:**
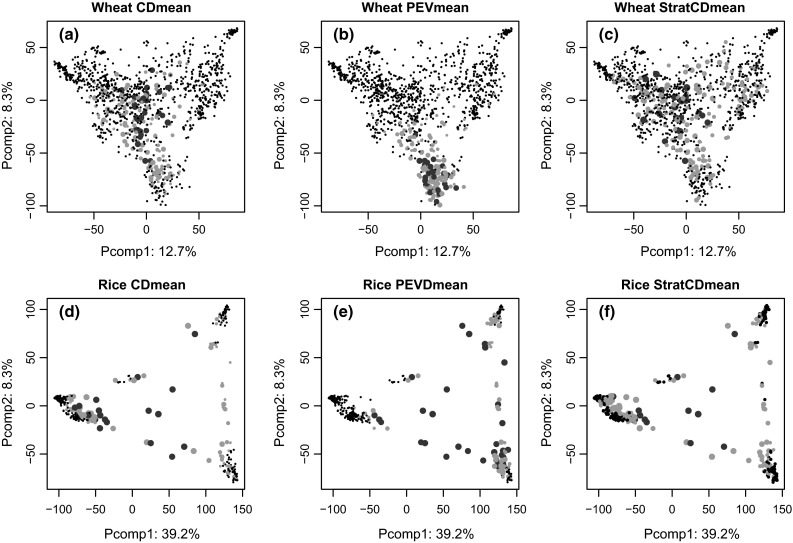
Genotypes selected from the optimization algorithm over the 50 run are plotted on the principal components analysis in wheat and rice germplasm. The genotypes were selected based on CDmean (**a**, **d**), PEVmean (**b**, **e**) and StratCDmean (**e**, **f**). *Green dots* represent the genotypes selected by the algorithm over the 50 runs. *Red dots* indicate those genotypes that were selected more than 15 and 27 times in wheat and rice germplasm, respectively (color figure online)

For both populations, Fig. 5 shows for the TRS size of 25, the PCA axes for the genotypes selected by the algorithms based on CDmean, PEVmean and stratCDmean methods. This figure illustrates the functional role of the algorithm in selecting the best genotypes to generate the optimized TRS as well as the variability of the panel captured by the TRS. In both populations, CDmean frequently selected most of the genotypes from the center of the PCs, and only rarely selected genotypes from the extremes of the clusters. This feature was observed more clearly for wheat than for rice (Figs. 3, 4, 5a, d). These patterns were stable across runs and traits. For the wheat dataset, most of the TRS genotypes selected using the PEVmean method were from the Elite Eastern cluster, with few genotypes from the center of the PCs (Fig. 5b). This pattern was also observed in the rice population, where PEVmean did not select genotypes from the *Temperate Japonica* cluster and more frequently selected genotypes from the *Indica/Aus* cluster (Fig. 5e). Although StratCDmean selected genotypes more disperse within clusters than other sampling algorithm, this was not reflected in an increase of the accuracies (Fig. 5c, f). Although, the algorithm forced CDmean to pick genotypes within clusters, most of the genotypes that were repeatedly selected tended to be from the center of the PCs in both wheat and rice populations.

### Relative phenotypic variance and accuracy

**Fig. 6:**
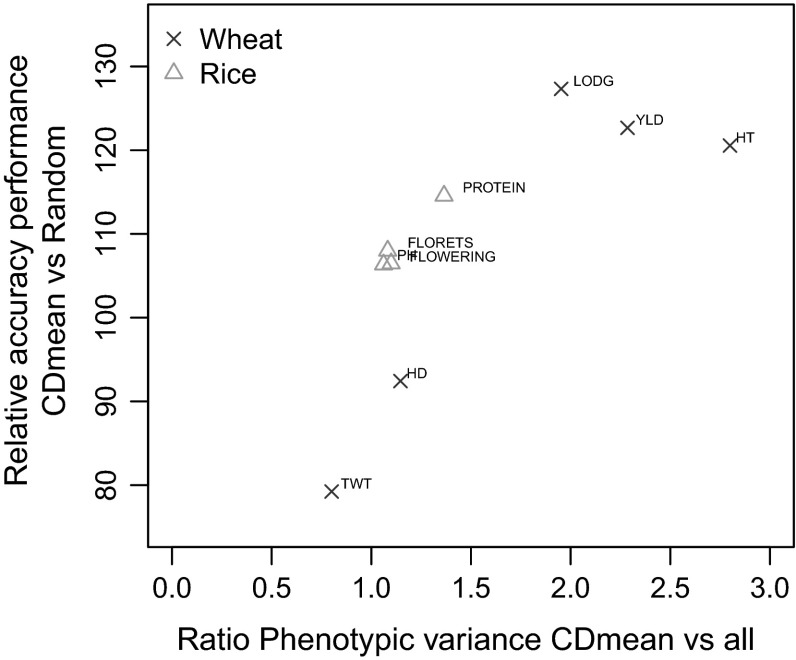
Relative performance of CDmean versus the random sampling method as a function of the ratio of the phenotypic variance between the top 40 most selected genotypes by CDmean and the overall phenotypic variance in wheat (*red-cross*) and rice (*blue-triangle*). Traits per germplasm are indicated in wheat as *YLD* yield, *TWT* test weight, *LODG* lodging, *HD* heading date, *HT* plant height and in rice as *FLORETS* florets per panicle, *FLOWERING* flowering time, *PH* plant height, *PROTEIN* protein content (color figure online)

Because of the different behavior of the test weight and heading date traits in wheat, we conducted additional analyses to determine the relationship between the phenotypic variance and accuracy. The ratio of the phenotypic variance of the genotypes most selected by CDmean and the total variance was plotted against the relative accuracy between CDmean and random sampling methods in a TRS size of 40 genotypes. If the value of the ratio between CDmean and the total variance is greater than 1.0, it means that extreme phenotypes are overrepresented in the TRS, while close-to average phenotypes are underrepresented. There was a positive overall relationship between the phenotypic variance captured by the TRS, and the relative accuracy of CDmean versus the random sampling method. Within the dataset the same relationship was observed more clearly in the wheat population than in rice. Figure 6 shows that CDmean only performed well when the ratio of the phenotypic variance of the TRS and the total phenotypic variance was greater than or equal to two. Yield, lodging and plant height were the only traits in the wheat germplasm, where CDmean performed better than random sampling. In the wheat dataset, CDmean did not perform well for test weight and heading date (Fig. 3c, d). For these traits, the accuracies were the lowest and CDmean did not capture a larger phenotypic variance. In the rice dataset, CDmean did not perform better than random sampling (Fig. 4). Here, as observed for wheat, rice traits were grouped together and showed the same positive relationship between the relative phenotypic variance and accuracy.

## Discussion

In a scenario where we have a diverse panel of genotypes that have been genotyped but not phenotyped, the first question that arises is how to select the best genotypes to create a TRS to build our statistical model for making predictions in the TS. The goal is to select the minimum number of genotypes that assure an optimal accuracy on the TS population. Several studies (Maenhout et al. 2010; Saatchi et al. 2011; Clark et al. 2011, 2012; Pszczola et al. 2012) and more recently by Guo et al. (2014) have highlighted the criteria to build an optimal TRS.

Rincent et al. (2012) developed algorithms to select an improved TRS that strategically sampled the genotypic space when developing training sets for genomic prediction. In this paper, our aim was to compare the performance of five algorithms, including the procedures from Rincent et al. (2012), in the presence of population structure using three different TRS optimization selection methods. These methods were tested on two different germplasm panels with different origins, different population structure effects, and in nine different traits with heritabilities ranging from 0.70 to 0.95 (Table 2). Our results indicated that the best selection criterion used to optimize the TRS was not consistent among populations. This seems to indicate that the interaction of trait architecture and population structure plays an important role in the optimization of the TRS. In six out of the nine traits studied in this analysis, the stratified sampling method showed higher accuracies than CDmean, PEVmean and StratCDmean, indicating that the degree of population structure is important in the design of the TRS. In populations with strong structure, as observed for the rice population, stratified sampling performed better than other methods (Fig. 4). In contrast, CDmean and StratCDmean showed better accuracies where population structure effects were mild, as observed for the wheat germplasm (Fig. 3). The similarity of accuracies for CDmean and StratCDmean can be explained by the fact that the contrasts used for both approaches to calculate the CDmean statistics were the same.

The divergence in selection method performance for test weight and heading date traits in wheat was unexpected and additional analyses were performed to explain the result. We found that these different results for test weight and heading date could be explained by the total phenotypic variance sampled for the trait (Fig. 6). For those traits, stratified sampling showed higher accuracies than CDmean, PEVmean and StratCDmean methods but was not different from random sampling. Our results in Fig. 6 indicated that, when CDmean captures most of the phenotypic variance the accuracies increased, as indicated for the traits yield, lodging and plant height. In contrast to this observation, the large genotypic variance obtained by CDmean does not always translate into a higher phenotypic variance ratio in the TRS. This might explain why CDmean performed poorly for test weight and heading date, because on average it produced TRS with reduced phenotypic variance compared to a random sampling. In addition, the lower phenotypic variance for test weight and heading date could be due to fewer genes affecting these traits in comparison to the other traits. These results seem to indicate that the best strategy may be to maximize the phenotypic variance captured by the TRS. In fact, recent studies (Jiménez-Montero et al. 2012; Boligon et al. 2012) have shown that strategies that maximize the phenotypic variance, through picking individuals from the two-tail distribution, are preferable to using genotypes with the largest or lowest phenotypic deviation. Empirical studies are needed to endorse the simulation results. Capturing most of the phenotypic variance in the training set seems to be key for optimal performance.

CDmean showed higher accuracies than PEVmean among traits and populations, with the only exception being for intermediate TRS set sizes for test weight for wheat (Fig. 3d). The optimal design for a TRS population for use in genomic prediction should minimize the relationship among genotypes in the TRS and maximize the relationship of the TS genotypes to the TRS. Consequently, the genotypes belonging to the TRS should not be closely related to each other but should be representative of the entire population. This is the main benefit to using the CDmean, because it takes into account the covariance among the candidate genotypes preventing the selection of closely related genotypes (Lalöe et al. 1993; Rincent et al. 2012). The CDmean algorithm most frequently selected genotypes situated near the center of the PCA under the effect of population structure, indicating that CDmean minimized the genetic distance to each cluster resulting in optimal performance when there was mild population structure. In contrast to the results found by Rincent et al. (2012), the CDmean method did not include all the extreme genotypes from each cluster. For example, in the wheat population the most frequent genotypes selected by CDmean belonged to the Ancestral Varieties (Fig. 5a).

The StratCDmean method was chosen to force the CDmean algorithm to select more extreme genotypes from different clusters. Although, StratCDmean improved the sampling of the extremes of the genotypes in different clusters, our results indicated that this strategy did not improve the accuracies of the predictions in either the rice or the wheat datasets. This could be due to the fact that the contrasts used in the CDmean and StratCDmean were the same.

CDmean and StratCDmean gave the highest accuracies for the smallest TRS size in both populations (Figs. 4, 5). This indicated that under the effect of population structure, CDmean and StratCDmean will perform better, on average, than the other methods, and therefore would be favored among these methods when the size of the TRS is small.

As observed by Rincent et al. (2012), the performance of the PEVmean in both populations revealed patterns similar to CDmean. One pitfall to using PEVmean to optimize the TRS is that, in contrast to CDmean, PEVmean selected a high number of related genotypes to create the TRS, which was not optimal (Fig. 5b, e). While accuracies between PEVmean and CDmean were not very different among traits and germplasm, the fact that it included more closely related genotypes would limit long-term gains from selection needed in plant breeding schemes (Jannink et al. 2010). Nevertheless, PEV is still an appropriate selection criterion for a measure of connectedness (Kennedy and Trus 1993). As shown in Fig. 5b, genotypes selected by PEVmean did not cover a wide genotypic space from the relationship matrix, but it selected a larger sample of Elite wheat genotypes.

The efficiency of the methods in terms of computational time also plays an important role in choosing a method for optimization. From the three methods used here, stratified sampling was the most efficient (less than a day), followed by StratCDmean (2 days), and CDmean (4 days). The fact that StratCDmean did not show large differences in accuracies in comparison with CDmean, and also improved the speed of the algorithm, made it more suitable than CDmean in the presence of population structure.

It is also important to note that the size of the CS can limit the use of CDmean. The algorithm requires the inversion of large matrices at each iteration to optimize the TRS, making it computationally intensive for large population CS sizes. For example in our study, the time to find the optimum took 50 % more time using the wheat dataset compared to rice. In addition, for stratified sampling and StratCDmean methods to be effective, a sufficient number of genotypes per cluster is required for the sampling algorithm. When the number of genotypes per cluster is too small, the stratified sampling is less useful.

TRS design for GS has attracted much attention in both animal and plant breeding in recent years because it is critical to the accuracy of the prediction models. However, less consideration has been given to the test population in the optimization process. We believe that the use of information from the test set could be valuable to improve accuracies of prediction models for TRS design. In this sense, an alternative to the maximization of the CDmean in the TRS could be the minimizing the PEVmean in the test set. Thus, the information about the test dataset could be used, while building the prediction model, by selecting the genotypes for the TRS that minimize the PEV of the test set.

In our optimization criteria, as well as in Rincent et al. (2012), the information from performance of relatives was incorporated through the use of a relationship matrix to calculate GEBVs. This is appropriate if major genes are not involved in the trait of interest. If the genetic distance based on genome-wide markers does not reflect the variability of the trait because major genes are involved, markers are not expected to be efficient for guiding the sampling of the TRS. If the optimal calibration set depends on the trait considered, this might be a problem for the implementation of GS in breeding programs because selection objectives usually involve multiple traits. Instead of using genomic prediction models for traits with major genes, it might be better to use models that include large effect loci as fixed effects in GS models. Studies have shown that including large effect loci in GS models can improve significantly the prediction accuracies. (Heslot et al. 2012; Gianola 2013; Bernardo 2014; Rutkoski et al. 2014). The information about the trait architecture learned from these models could be used in the future for developing new criteria for optimization. In addition, it should be mentioned that our results come from an additive genetic model and it might be worthwhile to explore the use of other models that can capture genetic effects such as epistasis and genotype-by-environment interaction. In this study, we only measure the effect of population structure on the optimization of the TRS, however some of the variation observed in our results could be due to other unmeasured features, because accuracies from prediction models depend on a complex network of different, interrelated factors.

We showed that population structure played an important role in the optimization of the TRS. When population structure effects are minor, CDmean performed better than other selection methods and captured most of the genetic variability for most traits in the TRS. This makes it suitable as an optimization criterion for long-term selection. However, under strong population structure stratified sampling performed better than CDmean, indicating that population structure must be evaluated before optimization to be sure the algorithm used does not reduce the phenotypic variation. Our results indicate that the overall optimization method works best when the trait under study is polygenic, because the genome-wide relationship measured by the **G** matrix captures the phenotypic relationship adequately. If the underlying genetic control of the trait is not polygenic, then the success of the training optimization techniques will similarly depend on whether or not the alleles of the trait are aligned with the overall structure. Stratified sampling is expected to perform best if the alleles controlling the traits are distributed according to the structure.

### **Author contributions**

J.-L.J. Contributed to experimental design and analysis and reviewed the manuscript. D.A. Contributed to algorithm development, analysis and reviewed of the manuscript. J.P. Contributed to design and development of genotyping-by-sequencing markers. N.H. Contributed to experimental design and reviewed the manuscript. M.E.S. Contributed to experimental design and analysis and reviewed the manuscript.

## Electronic supplementary material

Below is the link to the electronic supplementary material.

Supplementary material 1 (DOCX 187 kb). S1 Example of optimization of training population test for 100 genotypes by the stratified sampling algorithm (Method 1). The overall population size (1127) was randomly divided into a calibration set (627, CS) and test set (500, TS) with approximately same size. Cluster analysis was performed and a proportional stratified sampling algorithm, based on the sample size of the cluster was applied to build the TRS. Thirty-four genotypes from cluster 1 contribute to the total TRS, 29 from C2, 21 from C3 and 16 from C4. The sum of genotypes from every cluster will build the TRS. The entire process was repeated 50 times and the CS and TS individuals were recorded to use them as input for the other methods

Supplementary material 2 (DOCX 241 kb). S2 Example of optimization of training population test for 100 genotypes by coefficient of determination (CD), prediction error variance (PEV), and the random algorithm (Method 2). In method 2, the same genotypes chosen randomly by method 1 were used to build the CS (627) and TS (500). From the CS, a random sample of the targeted TRS, in this example 100 genotypes, was selected and the CD was calculated. The remaining genotypes create the remaining set (527, RS). Once the first CD is calculated, the CDmean algorithm is run and a new genotype will be accepted when the CDmean is increased. Iterations of the algorithm will stop when a CDmean maximum is reached. In the case of PEV, it will be accepted when the value of the PEVmean is lower than the initial one and the algorithm will stop when the PEVmean is no longer decreased. The entire process is repeated 50 times

Supplementary material 3 (DOCX 218 kb). S3 This method combines methods 1 and method 3 simultaneously. The same CS and TS from method 1 were used to build the TRS here. Cluster analysis was performed in the CS and a proportional stratified sampling was run for each cluster to generate the target size of the TRS. Next, the second method was applied here. For each cluster, the CDmean algorithm was applied. If any genotype increases the CDmean that genotype will remain in the TRS. Iterations will stop when a CDmean maximum is reached

Supplementary material 4 (DOCX 64 kb). S4: Percentage of variance explained by the structure in the wheat dataset. YLD, yield; TWT, test weight; LODG, lodging; HD, heading date; HT, plant height; FP, Florets per panicule; FT, flowering time; PH, plant height; PC; protein content. Df, degree of freedom; R^2^, proportion of the variance explained by the cluster

Supplementary material 5 (DOCX 58 kb). S5: Percentage of variance explained by the structure in the rice dataset. FP, Florets per panicule; FT, flowering time; PH, plant height; PC; protein content. Df, degree of freedom; R^2^, proportion of the variance explained by the cluster

Supplementary material 6 (DOCX 58 kb). S6: Accuracies mean percentage relative to random sampling in the wheat dataset. The mean of accuracies across sample size per trait and method was calculated and then compared to random sampling. Method with a positive value indicates on average, a better percentage of accuracy than random sampling. Negative values imply better performance of the random sampling method. i.e. 15.7 will indicate that for yield, CDmean performed on average 15.7 % better than random sampling. YLD, yield; TWT, test weight; LODG, lodging; HD, heading date; HT, plant height

Supplementary material 7 (DOCX 56 kb). S7: Accuracies mean percentage relative to random sampling in the rice dataset. The mean of accuracies across sample size per trait and method was calculated and then compared to random sampling. A method with a positive value indicates on average, a better percentage of accuracy than random sampling. Negative values imply better performance of the random sampling method. i.e. -1.35 will indicate that for florets per panicule, random sampling performed on average 1.35 % better than CDmean. FP, Florets per panicule; FT, flowering time; PH, plant height; PC; protein content
