# Supplementary figures and images for: Training set optimization under population structure in genomic selection
Source: Theor Appl Genet. 2014 Nov 1;128(1):145–58. doi: 10.1007/s00122-014-2418-4 (PMC4282691; doi:10.1007/s00122-014-2418-4)

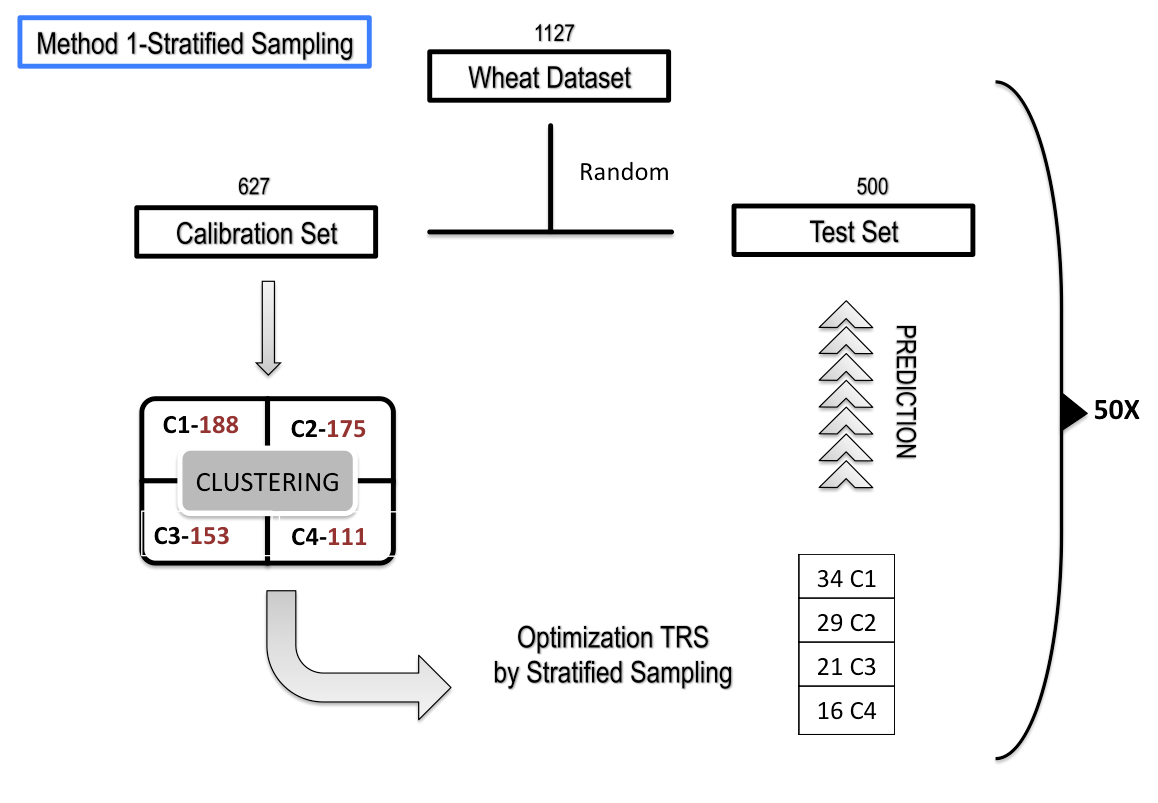

Supplement: Supplementary file 1 — Supplementary material 1 (DOCX 187 kb). S1 Example of optimization of training population test for 100 genotypes by the stratified sampling algorithm (Method 1). The overall population size (1127) was randomly divided into a calibration set (627, CS) and test set (500, TS) with approximately same size. Cluster analysis was performed and a proportional stratified sampling algorithm, based on the sample size of the cluster was applied to build the TRS. Thirty-four genotypes from cluster 1 contribute to the total TRS, 29 from C2, 21 from C3 and 16 from C4. The sum of genotypes from every cluster will build the TRS. The entire process was repeated 50 times and the CS and TS individuals were recorded to use them as input for the other methods [file 122_2014_2418_MOESM1_ESM.docx]

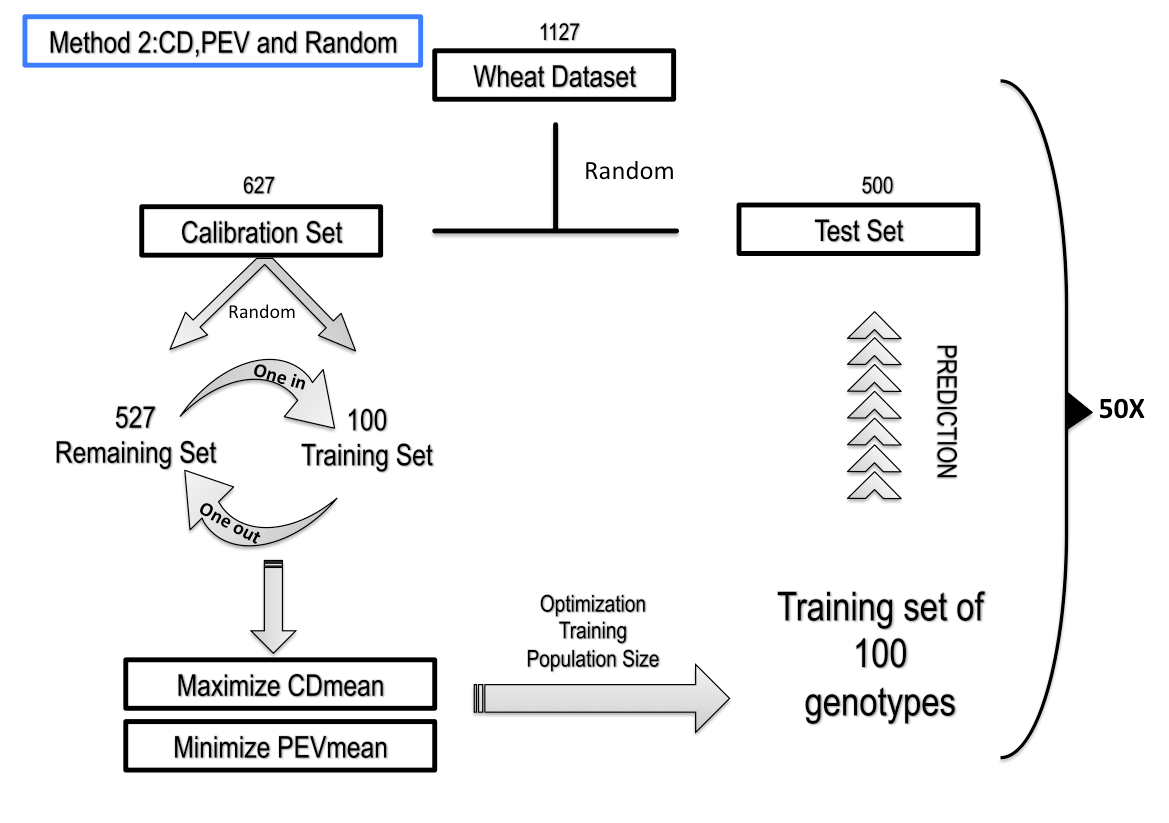

Supplement: Supplementary file 2 — Supplementary material 2 (DOCX 241 kb). S2 Example of optimization of training population test for 100 genotypes by coefficient of determination (CD), prediction error variance (PEV), and the random algorithm (Method 2). In method 2, the same genotypes chosen randomly by method 1 were used to build the CS (627) and TS (500). From the CS, a random sample of the targeted TRS, in this example 100 genotypes, was selected and the CD was calculated. The remaining genotypes create the remaining set (527, RS). Once the first CD is calculated, the CDmean algorithm is run and a new genotype will be accepted when the CDmean is increased. Iterations of the algorithm will stop when a CDmean maximum is reached. In the case of PEV, it will be accepted when the value of the PEVmean is lower than the initial one and the algorithm will stop when the PEVmean is no longer decreased. The entire process is repeated 50 times [file 122_2014_2418_MOESM2_ESM.docx]

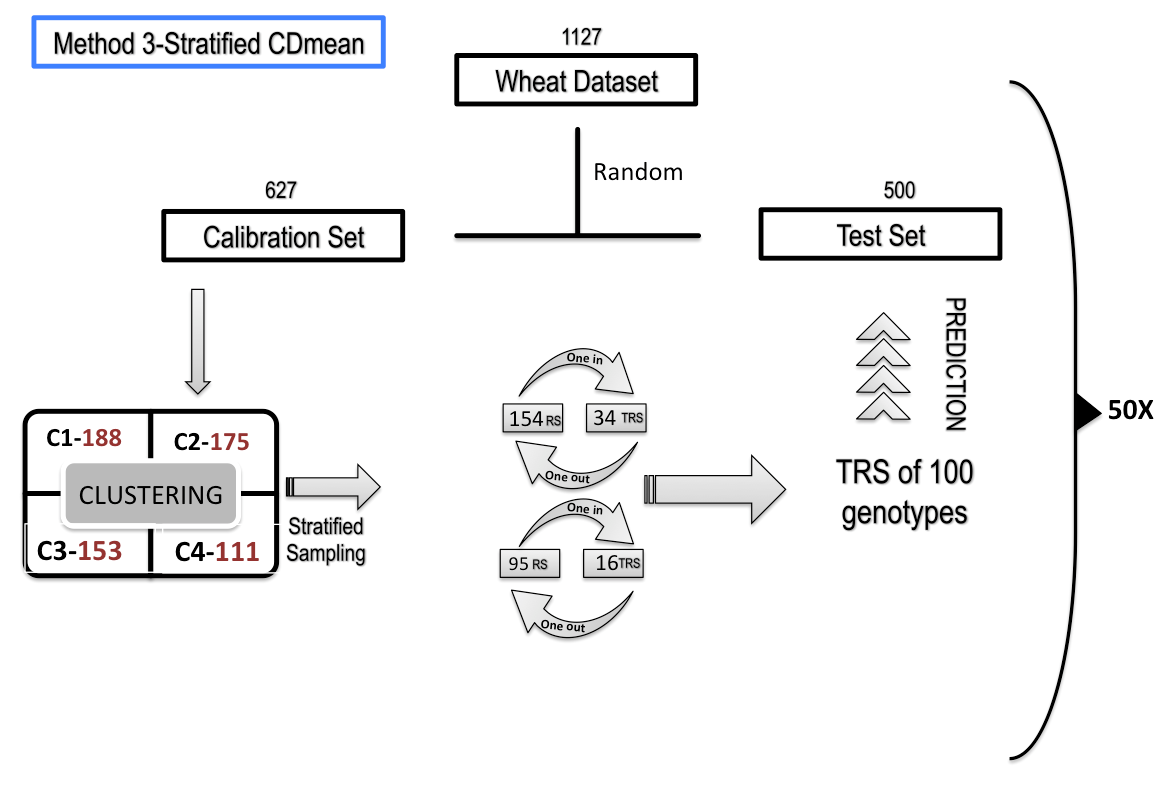

Supplement: Supplementary file 3 — Supplementary material 3 (DOCX 218 kb). S3 This method combines methods 1 and method 3 simultaneously. The same CS and TS from method 1 were used to build the TRS here. Cluster analysis was performed in the CS and a proportional stratified sampling was run for each cluster to generate the target size of the TRS. Next, the second method was applied here. For each cluster, the CDmean algorithm was applied. If any genotype increases the CDmean that genotype will remain in the TRS. Iterations will stop when a CDmean maximum is reached [file 122_2014_2418_MOESM3_ESM.docx]
