# Supplementary material for: Training set optimization under population structure in genomic selection
Source: Theor Appl Genet. 2014 Nov 1;128(1):145–58. doi: 10.1007/s00122-014-2418-4 (PMC4282691; doi:10.1007/s00122-014-2418-4)
Supplement: Supplementary file 4 — Supplementary material 4 (DOCX 64 kb). S4: Percentage of variance explained by the structure in the wheat dataset. YLD, yield; TWT, test weight; LODG, lodging; HD, heading date; HT, plant height; FP, Florets per panicule; FT, flowering time; PH, plant height; PC; protein content. Df, degree of freedom; R2, proportion of the variance explained by the cluster [file 122_2014_2418_MOESM4_ESM.docx]

| Trait | Source of Variation | Df | Sum Sq | Mean Sq | F value | Pr(>F) | R^2^(%) |
| --- | --- | --- | --- | --- | --- | --- | --- |
|  |  |  |  |  |  |  |  |
| YLD | Cluster | 3 | 1597837 | 532612 | 24.2 | 3.5 e^-15^*** | 6.1% |
|  | Residuals | 1121 | 24670035 | 22007 |  |  |  |
|  |  |  |  |  |  |  |  |
| TWT | Cluster | 3 | 40.0 | 13.3 | 22.6 | 3.2 e^-14^*** | 5.7% |
|  | Residuals | 1121 | 661 | 0.59 |  |  |  |
|  |  |  |  |  |  |  |  |
| LODG | Cluster | 3 | 1.76 | 0.588 | 9.08 | 6.1 e^-12^*** | 15.4% |
|  | Residuals | 1121 | 72.6 | 0.065 |  |  |  |
|  |  |  |  |  |  |  |  |
| HD | Cluster | 3 | 255 | 85.1 | 56.2 | 2.2 e^-20^*** | 13.0% |
|  | Residuals | 1121 | 1700 | 1.52 |  |  |  |
|  |  |  |  |  |  |  |  |
| HT | Cluster | 3 | 3242 | 1081 | 33.4 | 2.2 e^-16^*** | 8.2% |
|  | Residuals | 1121 | 36292 | 32.4 |  |  |  |
