# Supplementary material for: Training set optimization under population structure in genomic selection
Source: Theor Appl Genet. 2014 Nov 1;128(1):145–58. doi: 10.1007/s00122-014-2418-4 (PMC4282691; doi:10.1007/s00122-014-2418-4)
Supplement: Supplementary file 5 — Supplementary material 5 (DOCX 58 kb). S5: Percentage of variance explained by the structure in the rice dataset. FP, Florets per panicule; FT, flowering time; PH, plant height; PC; protein content. Df, degree of freedom; R2, proportion of the variance explained by the cluster [file 122_2014_2418_MOESM5_ESM.docx]

| Trait | Source of Variation | Df | Sum Sq | Mean Sq | F value | Pr(>F) | R^2^(%) |
| --- | --- | --- | --- | --- | --- | --- | --- |
|  |  |  |  |  |  |  |  |
| FP | Cluster | 2 | 3.21 | 1.60 | 17.8 | 4.2 e^-18^*** | 10.0% |
|  | Residuals | 354 | 31.9 | 0.09 |  |  |  |
|  |  |  |  |  |  |  |  |
| FT | Cluster | 2 | 9982 | 4991 | 42.5 | 2.2 e^-16^*** | 21.0% |
|  | Residuals | 354 | 41583 | 117 |  |  |  |
|  |  |  |  |  |  |  |  |
| PH | Cluster | 2 | 31565 | 15782 | 46.6 | 2.2 e^-16^*** | 18.6% |
|  | Residuals | 354 | 119835 | 338 |  |  |  |
|  |  |  |  |  |  |  |  |
| PC | Cluster | 2 | 29.7 | 14.87 | 21.8 | 1.1 e^-19^*** | 8.3% |
|  | Residuals | 354 | 241 | 0.682 |  |  |  |
