# Supplementary material for: Training set optimization under population structure in genomic selection
Source: Theor Appl Genet. 2014 Nov 1;128(1):145–58. doi: 10.1007/s00122-014-2418-4 (PMC4282691; doi:10.1007/s00122-014-2418-4)
Supplement: Supplementary file 6 — Supplementary material 6 (DOCX 58 kb). S6: Accuracies mean percentage relative to random sampling in the wheat dataset. The mean of accuracies across sample size per trait and method was calculated and then compared to random sampling. Method with a positive value indicates on average, a better percentage of accuracy than random sampling. Negative values imply better performance of the random sampling method. i.e. 15.7 will indicate that for yield, CDmean performed on average 15.7 % better than random sampling. YLD, yield; TWT, test weight; LODG, lodging; HD, heading date; HT, plant height [file 122_2014_2418_MOESM6_ESM.docx]

|  | CDmean (%) | PEVmean (%) | StratCDmean (%) | Stratified Sampling (%) |
| --- | --- | --- | --- | --- |
| YLD | 15.7 | 7.1 | 21.7 | 3.7 |
| TWT | -12.0 | -2.8 | -10.6 | -3.5 |
| LODG | 12.8 | -1.7 | 9.1 | 2.5 |
| HD | -11.7 | -13.9 | -14.6 | 1.6 |
| HT | 8.3 | 2.4 | 4.1 | 1.0 |
