# Supplementary material for: Training set optimization under population structure in genomic selection
Source: Theor Appl Genet. 2014 Nov 1;128(1):145–58. doi: 10.1007/s00122-014-2418-4 (PMC4282691; doi:10.1007/s00122-014-2418-4)
Supplement: Supplementary file 7 — Supplementary material 7 (DOCX 56 kb). S7: Accuracies mean percentage relative to random sampling in the rice dataset. The mean of accuracies across sample size per trait and method was calculated and then compared to random sampling. A method with a positive value indicates on average, a better percentage of accuracy than random sampling. Negative values imply better performance of the random sampling method. i.e. -1.35 will indicate that for florets per panicule, random sampling performed on average 1.35 % better than CDmean. FP, Florets per panicule; FT, flowering time; PH, plant height; PC; protein content [file 122_2014_2418_MOESM7_ESM.docx]

|  | CDmean (%) | PEVmean (%) | StratCDmean (%) | Stratified Sampling (%) |
| --- | --- | --- | --- | --- |
| FP | -1.35 | -6.78 | -0.93 | 6.11 |
| FT | -1.83 | -7.43 | -2.53 | 3.79 |
| PH | 0.32 | -0.11 | -2.55 | 3.01 |
| PC | -2.57 | -5.54 | -3.82 | 6.97 |
